# Genome insights of *Enterococcus raffinosus* CX012922, isolated from the feces of a Crohn’s disease patient

**DOI:** 10.1186/s13099-021-00468-8

**Published:** 2021-12-07

**Authors:** Hailan Zhao, Yao Peng, Xunchao Cai, Yongjian Zhou, Youlian Zhou, Hongli Huang, Long Xu, Yuqiang Nie

**Affiliations:** 1 Department of Gastroenterology, the Second Affiliated Hospital, School of Medicine, South China University of Technology, Guangzhou, 510006 Guangdong People’s Republic of China; 2 Department of Gastroenterology, Guangzhou Digestive Disease Center, Guangzhou First People’s Hospital, Guangzhou, 510180 Guangdong People’s Republic of China; 3 Department of Gastroenterology and Hepatology, Shenzhen University General Hospital, Shenzhen, 518071 Guangdong People’s Republic of China

**Keywords:** *Enterococcus*, Megaplasmid, Antibiotic resistance genes, Virulence factor, Toxin-antitoxin system

## Abstract

**Background:**

*Enterococcus raffinosus* is one of the *Enterococcus* species that often cause nosocomial infections. To date, only one *E. raffinosus* genome has been completely assembled, and the genomic features have not been characterized. Here, we report the complete genome sequence of the strain CX012922, isolated from the feces of a Crohn’s disease patient, and perform a comparative genome analysis to the relevant *Enterococcus* spp. strains in silico.

**Results:**

*De novo* assembly of the sequencing reads of the strain CX012922 generated a circular genome of 2.83 Mb and a circular megaplasmid of 0.98 Mb. Phylogenomic analysis revealed that the strain CX012922 belonged to the *E. raffinosus* species. By comparative genome analysis, we found that some strains previously identified as *E. raffinosus* or *E. gilvus* should be reclassified as novel species. Genome islands (GIs), virulence factors, and antibiotic genes were found in both the genome and the megaplasmid, although pathogenic genes were mainly encoded in the genome. A large proportion of the genes encoded in the megaplasmid were involved in substrate utilization, such as raffinose metabolism. Giant megaplasmids (~1 Mb) equipped with toxin-antitoxin (TA) systems generally formed symbiosis relationships with the genome of *E. raffinosus* strains.

**Conclusions:**

*Enterococcus* spp. have a higher species-level diversity than is currently appreciated. The pathogenicity of *E. raffinosus* is mainly determined by the genome-encoded virulence factors, while the megaplasmid broadens the gene function pool. The symbiosis between the genome and the megaplasmids endows *E. raffinosus* with large genomic sizes as well as versatile gene functions, especially for their colonization, adaptation, virulence, and pathogenesis in the human gut.

**Supplementary Information:**

The online version contains supplementary material available at 10.1186/s13099-021-00468-8.

## Background

*Enterococcus* is a Gram-positive and lactic acid bacteria of the phylum Firmicutes. This bacteria is widely distributed in the human body and has been frequently isolated, especially from the human gut. The dominant species of this genus, such as *E. faecalis* (90-95%) and *E. faecium* (5-10%), are common commensals in the gut. However, other species, such as *E. casseliflavus*, *E. gallinarum*, and *E. raffinosus*, can cause human disease [1, 2]. Among them, *E. raffinosus*, a non-motile, catalase-negative, raffinose-positive, and facultative anaerobe bacterium [3], is the leading cause of nosocomial infections due to its widespread antibiotic and multidrug resistance [4, 5]. For example, glycopeptide-resistant (e.g., vancomycin-teicoplanin dually resistant) *E. raffinosus* strains have been isolated from inpatient samples presenting in nosocomial outbreaks [6–8]. Although increased numbers of enterococci have been observed in both ulcerative colitis (UC) and Crohn’s disease (CD), the effects of increasing enterococci on the origin or progress of IBD have yet to be determined [9]. In our pre-study, *Enterococcus* spp. strains were found isolated from all IBD patients, among which *E. raffinosus* strains were frequently isolated from CD patients (5/8) but not from UC patients (0/14) (data not shown), implying potential relations between this species and CD. Therefore, further studies of *E. raffinosus* isolated from the feces of CD patients could help illuminate the relations between them. Besides, although the first complete genome sequence of this species was recently published in the genome database of NCBI in 2021, the genomic feature of this species has not been clarified or reported. Here, we isolated an *E. raffinosus* strain CX012922 from the feces of a young female CD patient and *de novo* assembled the complete genome using Illumina and Nanopore sequencing reads. Whole-genome sequence-based taxonomy identification and comparative genome analysis were then performed to clarify the genome function on virulence, adaptation, and pathogenic effects.

## Materials and methods

### Strain isolation and characterization

Fresh fecal samples were collected from a 25-year-old Chinese woman with active CD from Guangdong (China) who suffered from chronic and relapsing abdominal pain and diarrhea. About one gram of fresh feces was added to a 50 mL conical tube containing 10 mL of sterile PBS, which was then thoroughly vortexed for 5 min and left to settle for 5 min. The feces suspension was further transferred to blood culture bottles (BD, BACTECTM Lytic/10 Anaerobic/F Culture Vials, America) supplemented with 5 mL sterile sheep blood and rumen fluid (ELITE-MEDIA, Shanghai, China). Bottles were incubated under aerobic or anaerobic conditions at 37 °C for 30 days, according to Lagier´s and Ruifu Yang’s culturomics strategy [10, 11]. Then, aliquots of 1 mL suspension were sterilely aspirated from the incubated culture and transferred to a 15 mL falcon tube. Serial dilution gradients of 10 to 10^12^ were then prepared using 10 as the dilution factor and sterile PBS as the diluent. Finally, 100 µL of each dilution was plated evenly on the broth agar plates to harvest colonies, and further purification was conducted by streaking. The harvested colonies were then enriched in Lysogeny Broth (LB) medium at 37 °C for 24 h and further identified by MALDI Biotyper RTC (Bruker Daltonics, Germany). Single colonies sufficiently grown were directly transferred to the MALDI Biotyper RTC 96 target spot, 1 µL Bruker bacterial test standard (BTS), and matrix solution were added sequentially to prepare the detection target. Taxa identification was carried out with the default settings. If the spectrum score was greater than or equal to 2.3, a high-confident taxa identification at the species level was suggested. After that, the 16S rRNA sequence of one strain CX012922, identified as *Enterococcus* sp., was obtained by PCR using 8f/1492r primer pair and sent to Beijing Genomics institution (BGI) for Sanger sequencing. The 16S rRNA sequence was aligned using the NCBI nucleotide (nt) collection database for taxa identification, and species were determined with 100% sequence coverage and > 97% sequence identity.

### Genome sequencing and assembly

DNA extraction was performed using the TaKaRa MiniBEST Bacteria Genomic DNA Extraction Kit (Takara, Japan) according to the manufacturer’s instructions. The DNA quality was robustly checked using the Synergy HTX Multi-Mode Reader (BioTek, USA). Whole-genome sequencing was performed using the Nanopore PromethION platform at MAGIGENE company (Guangzhou, China) and Illumina NovaSeq platform at Novogene (Nanjing, China). The Nanopore PromethION library was constructed using the SQK-LSK109 kit (OxfordNanopore Technologies, UK) according to the manufacturer’s instructions. Sequencing and base calling were performed using MinKNOW v1.15.4 with the FLO-MINSP6 flow cell (Oxford Nanopore Technologies, UK). Low-quality reads (≤ Q7) were removed and then re-checked and filtered using NanoPlot v1.35.5 [12]. Illumina NovaSeq libraries were constructed with 350 bp insert size and sequenced using the PE150 strategy. Quality control of the raw reads was performed, including adapter trimming and low-quality reads removal (Phred score ≤ 20). The quality of the Illumina NovaSeq raw reads and clean reads was visualized using FastQC v0.11.8 (https://github.com/s-andrews/FastQC). The genome was then *de novo* assembled using the Unicycler v0.4.9b assembler [13] with the default hybrid assembly pipeline.

### Phylogenomic characterization and plasmid detection

Taxonomy assignment was further confirmed using the gtdbtk_wf workflow of GTDT-Tk [14] at the genomic level. Average nucleotide identity (ANI) between the phylogenomic close genomes was calculated using fastANI [15]. A phylogenomic tree based on whole-genome CDSs was constructed using CVTree3 [16]. The plasmid was predicted using three methods with default settings: the online tools PlasmidFinder v2.1 [17], based on replicon sequence identity; PlasForest v1.2 (https://github.com/leaemiliepradier/ PlasForest), and mlplasmids v2.1.0 (https://gitlab.com/sirarredondo/analysis_mlplasmids), based on machine learning from sequence homology and pentamer frequencies.

### Comprehensive genome annotation

Comprehensive gene prediction and functional genome annotation were performed using the NCBI Prokaryotic Genome Annotation Pipeline (PGAP) [18]. Functional genome annotation was further conducted with multiple databases, including Carbohydrate-Active enZYmes (CAZy), Cluster of Orthologous Groups (COG), and Kyoto Encyclopedia of Genes and Genomes (KEGG) using eggNOGMapper v2.1.5 [19]. Comprehensive genome analysis, including subsystem annotation, specialty genes identification (transporters, virulence factors, drug targets, antibiotic resistance genes (ARG), antimicrobial resistance genes (AMRG)), and phylogenetic analysis, were performed using Pathosystems Resource Integration Center v3.6.10 (PATRIC; https://www.patricbrc.org/). Pathogenicity to humans was predicted using PathogenFinder v1.1 (https://cge.cbs.dtu.dk/services/PathogenFinder/). Identification of genomic islands (GIs) was performed using IslandViewer 4 [20].

### Quality assurance

Before sequencing, a single colony of the strain CX012922 was repeatedly subcultured on broth agar plates to confirm the purity. Taxa identification was verified by both MALDI Biotyper RTC and full-length 16S rRNA sequence alignment, which determined that strain CX012922 belongs to the genus *Enterococcus* (Additional file 1: Tables S1, S2). The assembled genome sequence was further inputted into GTDB-Tk, which uses the gold standard (i.e., whole genome ANI) in the genomic era for taxonomy assignments.

## Results and discussion

### Genomic feature

**Fig. 1:**
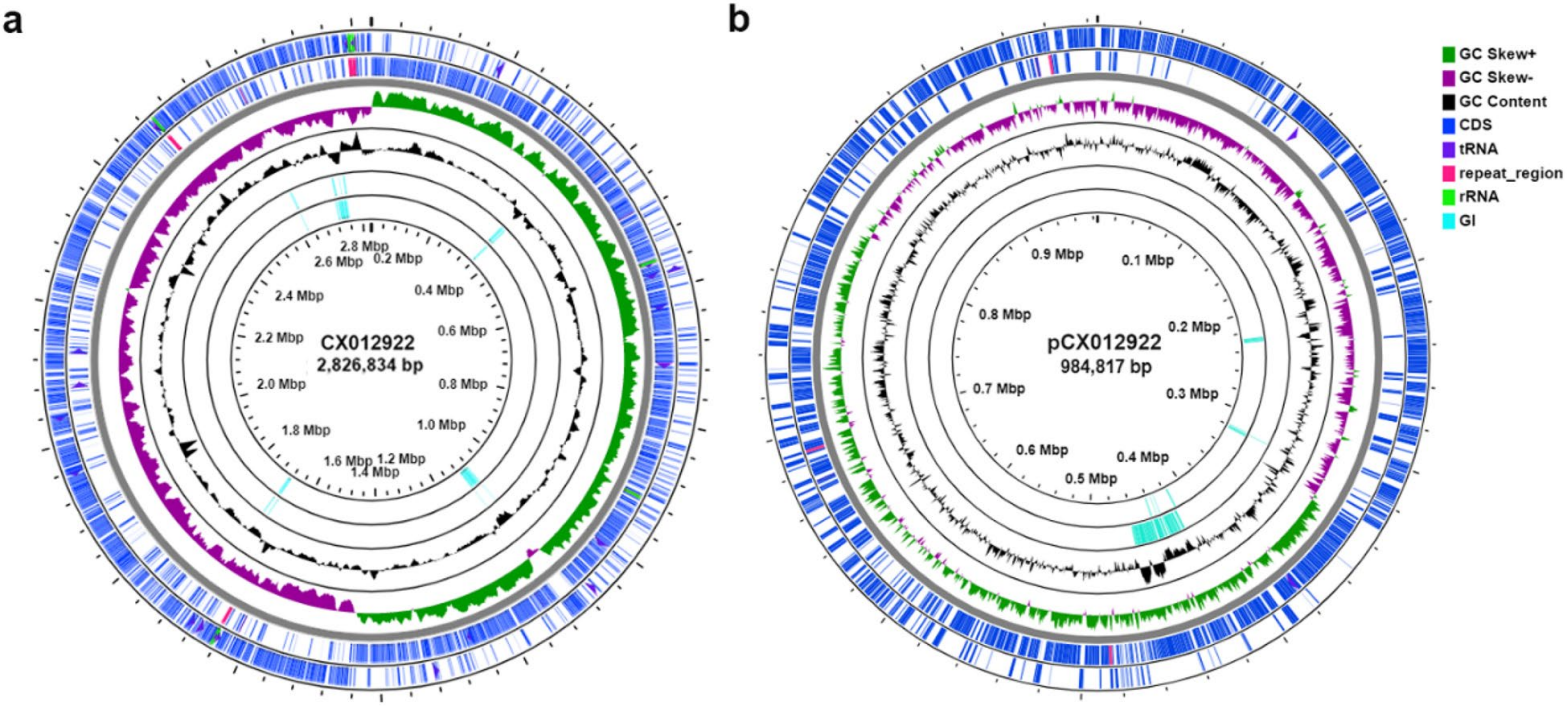
Circular display of the genome (**a**) and the plasmid (**b**) of *E. raffinosus* CX012922. From outer to inner rings, CDS on the forward strand, CDS on the reverse strand, GC skew, GC content, and genome islands. The RNAs and repeat sequence regions are displayed in the CDSs circles. GI represents genome islands

After conducting a hybrid assembly using Nanopore and Illumina reads, two circular contigs were obtained. The size of the bigger one is 2,826,834 bp, while the smaller one is 984,817 bp, completed with no N (Fig. 1). GTDB-Tk taxa identification workflow using the bigger contig as input sequence identified strain CX012922 as *E. raffinosus* (ANI >95), which was failed when referred to the smaller one as no bacteria marker genes were successfully extracted, suggesting that the smaller contig may be a megaplasmid. However, we found no existing plasmid replicon match to the smaller contig when running PlasmidFinder, further predictions based on machine learning methods (e.g., PlasForest and mlplasmids) identified it as a plasmid (data not shown). Besides, the genomic size of the strain CX012922 was found much smaller than most of the sequenced *E. raffinosus* strains (> 4 Mb, could be 4.7 Mb in some strain with undetermined plasmid sequences included) (https://www.ncbi.nlm.nih.gov/genome/browse/#!/prokaryotes/13061/), indicating that this species carries megaplasmids with novel replicons. The existence of the giant megaplasmid broadened the genome pool of *E. raffinosus*, enlarging their genomic size compared to that of some well-characterized *Enterococcus* commensals, such as *E. faecium* and *E. faecalis*. This megaplasmid might play a vital role in the virulence and host adaptation of *E. raffinosus*. In summary, the genome represents 74.16% of the entire genomic content, with an average G + C content of 39.43%, a total of 2,808 CDSs, 18 rRNAs, and 65 tRNAs (Fig. 1a). The circular megaplasmid constitutes a large proportion (25.84%) of the genomic content, from which 945 CDSs are predicted, and three tRNAs are annotated. Besides, five and three GIs are predicted in the genome and megaplasmid, respectively (Fig. 1b).

As the phylogenetically close strains (Additional file 1: Table S2) also carry megaplasmids, genomes assembled in complete level were collected for comparative analysis (Table 1). Similar to what we found in this study, the newly released complete genomic sequences of *E. raffinosus* F162_2 (8th July 2021) contain two circular megaplasmids of 1,203,089 bp and 38,224 bp: the total length of the megaplasmids accounts for 28.76% of the genomic content (Table 1). Furthermore, complete genome characteristics, such as genome size, genome proportion, rRNA counts, and tRNA counts of the strain *E. gilvus* CR1 showed high similarity to the *E. raffinosus* strains (Table 1). Interestingly, the giant megaplasmids pCX012922 and pF162_2_1 from the strain CX012922 and F162_2 respectively showed high sequence similarity (Additional file 1: Fig. S1). On the other hand, megaplasmids between species *E. raffinosus*, *E. gilvus*, and *E. avium* showed highly divergent features in length, G + C content, and CDS counts (Table 1). Although the genome size of *E. raffinosus* and *E. gilvus* is smaller than that of *E. avium* (Table 1), the gene pool of the former two species could be broadened by the accessory genes that reside in the megaplasmids. These results imply that megaplasmids are commonly carried by *E. raffinosus* strains and their relatives, and the sequence conservation of giant megaplasmids within *E. raffinosus* may bring them the capacity to colonize or adapt the host environments.

**Table 1:** Complete genome features of the strains phylogenetically close to CX012922

Strain	Accession	Type	Length	GC%	CDS	rRNA	tRNA	Other RNA	Genome proportion (%)
*E. raffinosus* CX012922	CP081846.1	Chromosome	2,826,834 bp	39.4	2,808	18	65	4	74.16
pCX012922	CP081847.1	Plasmid	984,817 bp	40.0	945	–	3	–	
*E. raffinosus* F162_2	CP072888.1	Chromosome	3,032,004 bp	39.5	2,955	18	68	4	71.24
pF162_2_1	CP072889.1	Plasmid	1,186,145 bp	39.9	1,107	–	3	–	
pF162_2_2	CP072890.1	Plasmid	37,686 bp	39.9	48	–	–	–	
*E. gilvus* CR1	CP030932.1	Chromosome	2,863,043 bp	41.9	2,805	18	65	4	72.57
pCR1A	CP030933.1	Plasmid	919,333 bp	42.9	869	–	–	–	
pCR1B	CP030934.1	Plasmid	80,244 bp	35.0	86	–	–	–	
pCR1C	CP030935.1	Plasmid	82,704 bp	36.8	80	–	–	–	
*E. avium* G-15	AP019814.1	Chromosome	3,623,727 bp	39.7	3,614	15	67	4	100
*E. avium* FDAARGOS_184	CP024590.1	Chromosome	3,723,378 bp	38.5	3,670	15	66	4	100
*E. avium* 352	CP034169.1	Chromosome	4,794,392 bp	39.0	4,761	18	70	4	98.20
Unnamed	CP034168.1	Plasmid	87,704 bp	35.5	97	–	–	–	

### Phylogenomic characterization

**Fig. 2:**
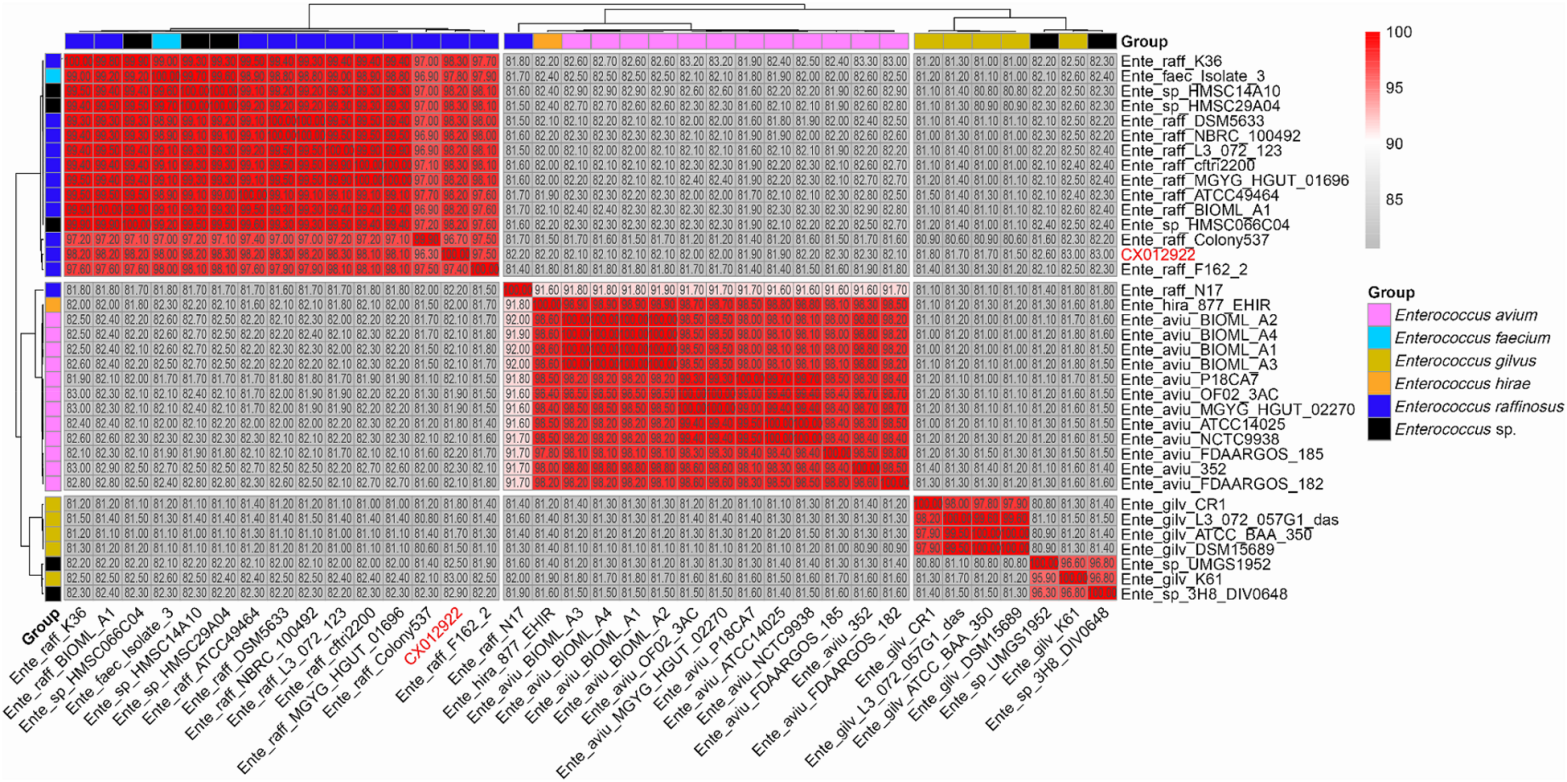
Phylogenomic characterization of the closely related *Enterococcus* spp. using ANI. Ente_raff, Ente_aviu, Ente_gilv, Ente_hira and Ente_sp represent *E. raffinosus*, *E. avium*, *E. gilvus*, *E. hirae* and *Enterococcus* sp. respectively

To further clarify the phylogenetic relationship between *E. raffinosus*, *E. gilvus*, *E. avium*, and others, the genome distance was calculated using the “Similar Genome Finder” function in PATRIC to find the phylogenetic relationships of the genomes close to the strain CX012922. The 36 most close genomes are presented in Additional file 1: Fig. S2. We found that these genomes could be clustered in three major groups, which displayed better resolution than MALDI Biotyper or 16S rRNA-based methods. Moreover, an identical clustering pattern was observed based on the WGS ANI method (Fig. 2). Interestingly, we found that the previous taxa assignments of some strains among these three groups should be reclassified as novel species according to the gold standard in the genomic era (ANI > 95%) [21]. For example, *E. faecium* Isolate_3 and *E. hirae* 877_EHIR should be reclassified as *E. raffinosus* and *E. avium*, respectively, while *E. raffinosus* N17 and *E. gilvus* K61 should be assigned as two novel species (Fig. 2). This clustering pattern could also be confirmed by the phylogenomic tree constructed from the whole genome sequences of these strains (Additional file 1: Fig. S3). To further verify these results, the “Similar Genome Finder” function implanted in PATRIC was used to calculate the genome distance between these two genomes (i.e., N17 and K61) and the public genomes. We found that no defined species had a genome distance lower than 0.05 to the genome of strains N17 and K61 (Additional file 1: Table S3), supporting our hypothesis that they should be redefined as novel species. These results suggest that *Enterococcus* spp. are much more diverse than we presently appreciate. Hence, efforts should be made to explore it, which would be helpful to explain their pathogenicity, virulence and adaptation capacities.

### Function genome annotation

Function genome annotation of strain CX012922 using RAST identified 737 and 148 genes in the genome and megaplasmid, respectively, belonging to the SEED subsystem (Additional file 1: Fig. S4). Among these genes, 37 in the genome while 11 in the megaplasmid belonged to the category “Virulence, Disease and Defense” (Additional file 1: Fig. S4), which included genes encoding metal resistance, antibiotic resistance, multidrug resistance efflux pumps, and bile hydrolysis (Additional file 1: Table S4). Interestingly, the antibiotic resistance coding genes are beta-lactamase but not the previously reported glycopeptide-resistant proteins [6–8], suggesting that vancomycin/teicoplanin resistance of this strain was acquired from the environments by horizontal gene transfer events. Besides, genes belonging to the category “Stress Response” and “Phages, Prophages, Transposable elements, Plasmids” were also carried by the genomes and plasmids (Additional file 1: Table S4). A total of 112 plasmid genes belonged to the category “Amino Acids and Derivatives” and “Carbohydrates”, which took a proportion of 75.68% to the subsystem annotated genes (Additional file 1: Fig. S4), implying that the megaplasmid may play vital roles in substrate metabolism and energy production. In particular, genes encoding raffinose metabolism such as K5P74_RS18540 (RafB, raffinose permease) and K5P74_RS18535, an exclusive biological trait in *E. raffinosus* and its relatives, were found to reside in the plasmid. Pathogenicity prediction based on PathogenFinder suggested strain CX012922 as a human pathogen and revealed that virulence factors primarily resided in the genome but not the megaplasmid (Additional file 1: Table S5), which was also observed in *E. raffinosus* F162_2 (data not shown). The virulence factors in the genome of CX012922 included ABC transporter homologs in *Listeria monocytogenes*, *E. faecalis*, and SSU ribosomal protein (S19P) homologs in *Streptococcus suis* (Additional file 1: Table S5). The above results suggested that the megaplasmid in *E. raffinosus* encodes functions not only related to “accessory functions” but also functions related to the basic metabolism, thus forming a symbiosis relationship with the genome. This kind of symbiosis or “plasmid addiction” has been found in many giant megaplasmids [22]. This relationship forms a toxin-antitoxin (TA) system that involves two components that are made by the plasmid: a toxin (long-lived) lethal to the host cell and an antidote (short-lived). Once the plasmid is lost, the cells die [23]. The comprehensive annotation of the megaplasmid sequences from strains CX012922 and F162_2 showed the presence of several TA systems, including the Type IV TA and Type II TA systems (Additional file 1: Table S6). Plasmid prediction of the *E. raffinosus* clade (Fig. 2) showed that all the *E. raffinosus* strains except Colony537 (genome length ~1 Mb, which may be the result of insufficient assembly), harbored at least one giant megaplasmid, with an average length proportion of 22.82% (10.91–32.56%) of the entire genomic content (Additional file 1: Table S7). Meanwhile, these plasmid sequences showed high sequence homology to that of pCX012922 and pF162_2_1, and toxin-antitoxin genes were also annotated in these predicted plasmids (data not shown). Consequently, the existence of plasmids may be a general genomic feature of *E. raffinosus*. The encoding function of the megaplasmids could broaden the metabolic capacities of *E. raffinosus* strains and help them survive in different environments.

## Conclusions

Here, we report the complete genome sequence of *E. raffinosus* CX012922, the general genome feature, phylogenomic traits, and function specialty were analyzed. The results displayed that the close phylogenetic species such as *E. raffinosus*, *E. gilvus*, and *E. avium* could be discriminated from each other in high resolution using WGS based analysis (i.e., ANI), and novel species were suggested to reclassify from some sequenced *Enterococcus* spp. Besides, the pathogenicity encoding genes of *E. raffinosus* CX012922 was observed mainly residing in the genome. Giant megaplasmids (~1 Mb) were found to be a general feature of *E. raffinosus*, which formed a symbiosis relationship with the genome and expanded the genome function pool to help the host adaptation. The results of this study broadened our knowledge of *E. raffinosus* at the genomic level and provided useful information for us to further explore their pathogenicity and adaptation mechanisms in the human body.

## Supplementary Information

**Additional file 1.**

## Data Availability

Raw reads, as well as de novo assembled draft genome sequence of strain CX012922 were submitted to GenBank and the Sequence Read Archive data base of the National Center for Biotechnology Information (NCBI), available under the BioProject accession PRJNA756165 and PRJNA756411, respectively. The complete genome sequence and the plasmid sequence were deposited in the genome database of GenBank, available under the accession number CP081846 and CP081847, respectively.

## References

[1] Gilmore MS, Clewell DB, Courvalin P, Dunny GM, Murray BE, Rice LB (2002). The enterococci: pathogenesis, molecular biology, and antibiotic resistance.

[2] Arias CA, Murray BE (2012). The rise of the *Enterococcus*: beyond vancomycin resistance. Nat Rev Microbiol..

[3] Collins MD, Facklam RR, Farrow JA, Williamson R (1989). *Enterococcus raffinosus* sp. nov., *Enterococcus solitarius* sp. nov. and *Enterococcus pseudoavium* sp. nov. FEMS Microbiol Lett.

[4] Choi HE, Lee JH, Sim YJ, Jeong HJ, Kim GC (2021). Predictors of prolonged vancomycin-resistant enterococci colonization in acute stroke patients admitted to an intensive care unit: A retrospective cohort study. Medicine (Baltimore).

[5] Santimaleeworagun W, Changpradub D, Hemapanpairoa J, Thunyaharn S. Optimization of linezolid dosing regimens for treatment of vancomycin-resistant enterococci infection. Infect Chemother. 2021. 10.3947/ic.2021.0034 PMC8511381 34405596

[6] Mathur P, Hollowoa B, Lala N, Thanendrarajan S, Matin A, Kothari A (2017). *Enterococcus raffinosus* infection with atypical hemolytic uremic syndrome in a multiple myeloma patient after autologous stem cell transplant. Hematol Rep.

[7] Jolivet S, Fines-Guyon M, Nebbad B, Merle D, Le Pluart C, Brun-Buisson JW (2016). First nosocomial outbreak of *vanA*-type vancomycin-resistant *Enterococcus raffinosus* in France. J Hosp Infect.

[8] Samuel J, Coutinho H, Galloway A, Rennison ME, Kaufmann, Neil W (2008). Glycopeptide-resistant *Enterococcus raffinosus* in a haematology unit: an unusual cause of a nosocomial outbreak. J Hosp Infect.

[9] Růžičková M, Vítězová, Kushkevych I (2020). The characterization of *Enterococcus* genus: resistance mechanisms and inflammatory bowel disease. Open Med-warsaw.

[10] Lagier JC, Dubourg G, Million M, Cadoret F, Bilen M, Fenollar F (2018). Culturing the human microbiota and culturomics. Nat Rev Microbiol.

[11] Chang Y, Hou F, Pan Z, Huang ZY, Han N, Lei B, Deng HM (2019). Optimization of culturomics strategy in human fecal samples. Front Microbiol.

[12] De Coster W, D’Hert S, Schultz DT, Cruts M, Van Broeckhoven C (2018). NanoPack: visualizing and processing long-read sequencing data. Bioinformatics.

[13] Wick RR, Judd LM, Gorrie CL, Holt KE (2017). Unicycler: Resolving bacterial genome assemblies from short and long sequencing reads. PLoS Comput Biol.

[14] Parks DH, Chuvochina M, Waite DW, Rinke C, Skarshewski A, Chaumeil PA (2018). A standardized bacterial taxonomy based on genome phylogeny substantially revises the tree of life. Nat Biotechnol.

[15] Jain C, Rodriguez-R LM, Phillippy AM, Konstantinidis KT, Aluru S (2018). High throughput ANI analysis of 90 K prokaryotic genomes reveals clear species boundaries. Nat Commun.

[16] Zuo G, Hao B (2015). CVTree3 web server for whole-genome-based and alignment-free prokaryotic phylogeny and taxonomy. Genom Proteom Bioinf.

[17] Carattoli A, Zankar E, García-Fernández A, Voldby LM, Lund O, Villa L (2014). In silico detection and typing of plasmids using PlasmidFinder and plasmid multilocus sequence typing. Antimicrob Agents CH.

[18] Tatiana T, Michael DC, Azat B, Vyacheslav C, Nawrocki EP, Zaslavsky L (2016). NCBI prokaryotic genome annotation pipeline. Nucleic Acids Res.

[19] Jaime HC, Kristoffer F, Pedro CL, Damian S, Juhl JL, Von Mering C (2016). Fast genome-wide functional annotation through orthology assignment by eggNOG-Mapper. Mol Biol Evol.

[20] Bertelli C, Laird MR, Williams KP, Lau BY, Hoad G, Simon Fraser University Research Computing Group (2017). IslandViewer 4: expanded prediction of genomic islands for larger-scale datasets. Nucleic Acids Res.

[21] Chun J, Oren A, Ventosa A, Christensen H, Arahal DR, da Costa MS (2018). Proposed minimal standards for the use of genome data for the taxonomy of prokaryotes. Int J Syst Evol Microbiol.

[22] Meinhart A, Alonso JC, Strater N, Saenger W (2003). Crystal structure of the plasmid maintenance system epsilon/zeta: functional mechanism of toxin zeta and inactivation by epsilon 2 zeta 2 complex formation. Proc Natl Acad Sci USA.

[23] Saramago M, Bárria C, Arraiano CM, Domingues S (2015). Ribonucleases, antisense RNAs and the control of bacterial plasmids. Plasmid.

