# Supplementary material for: Genome insights of Enterococcus raffinosus CX012922, isolated from the feces of a Crohn’s disease patient
Source: Gut Pathog. 2021 Dec 7;13:71. doi: 10.1186/s13099-021-00468-8 (PMC8650288; doi:10.1186/s13099-021-00468-8)
Supplement: Supplementary file 1 — Additional file 1. [file 13099_2021_468_MOESM1_ESM.pdf]

Supplementary materials for

Genome insights of *Enterococcus raffinosus* CX012922, isolated from the feces of a Crohn's disease patient

Hailan Zhao[1,†], Yao Peng[3,†], Xunchao Cai[3], Yongjian Zhou[1,2], Youlian Zhou[1,2], Hongli Huang[1,2], Long Xu[3,*], Yuqiang Nie[1,2,*]

[1]Department of Gastroenterology, the Second Affiliated Hospital, School of Medicine, South China University of Technology, Guangzhou, Guangdong, 510006, P.R. China

[2]Department of Gastroenterology, Guangzhou Digestive Disease Center, Guangzhou First People's Hospital, Guangzhou, Guangdong, 510180, P.R. China

[3]Department of Gastroenterology and Hepatology, Shenzhen University General Hospital, Shenzhen, Guangdong, 518071, P. R. China

[†]These authors contribute equally to this work.

[*]Co-corresponding author:

Tel: +86-020-26558094; E-mail address: eynieyuqiang@scut.edu.cn (Yuqiang Nie);

Tel: +86-755-21839136; E-mail address: longxu1012@szu.edu.cn (Long Xu).

Submitted to *Gut Pathogens*

The supplementary information contains seven tables and four figures.

Table Captions and Figure Legends

Table S1 Taxa identification using MALDI Biotyper RTC

Table S2 Top ten BLAST hits of 16S rRNA sequence of strain CX012922 against the NCBI-nt database

Table S3 Genome distance estimated top five close genomes to the that of strain N17 and K61

Table S4 The genome and plasmid genes related to pathogenicity, virulence and adaptation

Table S5 Pathogenicity prediction of the genome

Table S6 The toxin-antitoxin systems in the *E. raffinosus* mega-plasmids

Table S7 The predicted plasmid contigs in the genomes of *E. raffinosus* using three methods

Figure S1. Multiple collinearity between the giant mega-plasmids. Ente_raff_pCX012922.fasta, Ente_raff_pF162_2_1.fasta and Ente_gilv_pCR1A.fasta represent giant mega-plasmids from *E. raffinosus* CX012922, *E. raffinosus* F162_2 and *E. gilvus* CR1 respectively.

Figure S2. Heatmap displaying the genome distance between the phylogenetically close *Enterococcus* spp. Color bar in the right indicates the genome distance calculated using the "Similar Genome Finder" function in PATRIC.

Figure S3. Phylogenomic tree constructed from the whole genome sequences of the strains phylogenetic close to strain CX012922.

Figure S4. The genome and plasmid genes annotated to SEED subsystem functions.

Table S1 Taxa identification using MALDI Biotyper RTC

NO	Matched Pattern	Score	Reliability Level
1	*Enterococcus raffinosus* DSM 5633^T	1.82	Genus
2	*Enterococcus raffinosus* LMG 12172	1.77	Genus
3	*Enterococcus raffinosus* BK847	1.702	Genus
4	*Enterococcus avium* DSM20063	1.647	Unreliable
5	*Enterococcus gilvus* 6760	1.582	Unreliable
6	*Enterococcus gilvus* CCUG 60355	1.561	Unreliable
7	*Enterococcus gilvus* DSM 15689^T	1.558	Unreliable
8	*Enterococcus avium* LMG 22765	1.528	Unreliable
9	*Enterococcus avium* CCUG 44888	1.516	Unreliable
10	*Enterococcus avium* DSM 20679^T	1.464	Unreliable

The meaning of score is interpretated as follows: a. higher than 2.300, high confident taxa identification at species level; b. 2.000~2.299, high confident taxa identification at genus level, possible taxa identification at species level; 1.700~1.999, possible taxa identification at genus level; c. lower than 1.700, unreliable taxa identification.

Table S2 Top ten BLAST hits of 16S rRNA sequence of strain CX012922 against the NCBI-nt database

Strain	Scientific Name	Score	Total Score	Query Cover	E-value	Identity	Accession Length	Accession
F162_2	*Enterococcus raffinosus*	2874	17235	100%	0	99.94	3032004	CP072888.1
Colony537	*Enterococcus raffinosus*	2867	11464	100%	0	99.87	1007285	CP078553.1
CR1	*Enterococcus gilvus*	2863	17171	100%	0	99.81	2863043	CP030932.1
G-15	*Enterococcus avium*	2857	14267	100%	0	99.74	3623727	AP019814.1
FDAARGOS_184	*Enterococcus avium*	2852	14234	100%	0	99.68	3723378	CP024590.1
352	*Enterococcus avium*	2846	17080	100%	0	99.62	4794392	CP034169.1
E11	*Enterococcus raffinosus*	2819	2819	98%	0	99.80	4258	MK322666.1
LMG 12999	*Enterococcus raffinosus*	2817	2817	99%	0	99.36	1893	AJ301838.1
NA	*Enterococcus avium*	2808	2808	98%	0	99.55	1544	DQ779961.1
NA	*Enterococcus avium*	2808	2808	99%	0	99.35	1546	AY442814.1

The genome distance $D \approx$ 1-ANI, which means that $D \leqslant 0.05$ equates to an ANI $\geqslant$ 95 %. Thus, $D = 0.05$ could be the cut-off to cluster genomes in the same species [1]. Apparently, strain K61 together with strains *Enterococcus* sp. 3H8_DIV0648, *Enterococcus* sp. 190-7 and uncultured *Enterococcus* sp. UMGS1952 should defined as novel species in the genus *Enterococcus*, strain N17 alone should be defined as another novel species.

Table S3 Genome distance estimated top five close genomes to the that of strain N17 and K61

Genome ID	Genome Name	Isolation Source	Host Name	Genome Distance (*D*)	P Value
**K61**					
1834178	*Enterococcus* sp. 3H8_DIV0648	feces	Bird	0.027689	0
2559929	*Enterococcus* sp. 190-7	traditional fermented foods and other habitats	NA	0.029995	0
167972.4	uncultured *Enterococcus* sp. UMGS1952	human gut	NA	0.041569	0
1761783	*Enterococcus* sp. kppr-6	NA	NA	0.151946	1.25E-89
33945.48	*Enterococcus avium* BIOML-A4	fecal material	Human, Homo sapiens	0.154223	5.02E-85
**N17**					
33945.14	*Enterococcus avium* LC0559/18	stool	Human, Homo sapiens	0.066265	0
33945.8	*Enterococcus avium* FDAARGOS_182	abscess	Human, Homo sapiens	0.069006	0
2315837	*Enterococcus* sp. T0101B.F-10	stool	Human, Homo sapiens	0.069006	0
1715012	*Enterococcus* sp. HMSC072H05	urine	Human, Homo sapiens	0.069323	0
1715019	*Enterococcus* sp. HMSC064A12	sputum	Human, Homo sapiens	0.069644	0

Table S4 The genome and plasmid genes related to pathogenicity, virulence and adaptation

Subsystem Feature	Genome	Plasmid
**Virulence, Disease and Defense**	**37**	**11**
Resistance to antibiotics and toxic compounds	27	11
Invasion and intracellular resistance	10	0
**Phages, Prophages, Transposable elements, Plasmids**	**5**	0
Phages, Prophages	5	0
**Stress Response**	**25**	1
Osmotic stress	10	1
Oxidative stress	12	0
Detoxification	1	0
Stress Response - no subcategory	2	0
**Motility and Chemotaxis**	0	0

Table S5 Pathogenicity prediction of the genome

No.	Input Sequence	Accession [a]	Organisms	Class	Protein function	Protein ID [b]	Identity (%)	Matched Family
1	1_340 # 348783 # 349718 # 1 # ID=1_340;partial=00;start_type=ATG;rbs_motif=AGGA;rbs_spacer=5-10bp;gc_cont=0.379	CP001175	*Listeria monocytogenes* HCC23, complete genome.	Bacillales	ABC transporter, ATP-binding protein	ACK40303	84.89	Pathogenic Families
2	1_422 # 429481 # 430398 # -1 # ID=1_422;partial=00;start_type=ATG;rbs_motif=AGGAG;rbs_spacer=5-10bp;gc_cont=0.417	AE016830	*Enterococcus faecalis* V583, complete genome.	Lactobacillales	ABC transporter, ATP-binding protein	AAO82881	85.25	Pathogenic Families
3	1_421 # 428718 # 429488 # -1 # ID=1_421;partial=00;start_type=ATG;rbs_motif=GGAGG;rbs_spacer=5-10bp;gc_cont=0.440	AE016830	*Enterococcus faecalis* V583, complete genome.	Lactobacillales	ABC transporter, permease protein	AAO82880	88.67	Pathogenic Families
4	1_196 # 196713 # 196991 # 1 # ID=1_196;partial=00;start_type=ATG;rbs_motif=AGGAGG;rbs_spacer=5-10bp;gc_cont=0.380	CP000407	*Streptococcus suis* 05ZYH33, complete genome.	Lactobacillales	SSU ribosomal protein S19P	BAC14406	91.3	Pathogenic Families
5	1_191 # 193898 # 194206 # 1 # ID=1_191;partial=00;start_type=ATG;rbs_motif=AGGAGG;rbs_spacer=5-10bp;gc_cont=0.388	CP000922	*Anoxybacillus flavithermus* WK1, complete genome.	Bacillales	Ribosomal protein S10	ACJ32488	84.31	Non-pathogenic Families
6	1_1181 # 1205885 # 1206160 # 1 # ID=1_1181;partial=00;start_type=ATG;rbs_motif=AGGAGG;rbs_spacer=11-12bp;gc_cont=0.431	CP001084	*Lactobacillus casei* str. Zhang, complete genome.	Lactobacillales	nucleoid DNA-binding protein	ADK18601	87.91	Non-pathogenic Families

[a]NCBI genome accession ID

[b]NCBI protein ID

Table S6 The toxin-antitoxin systems in the *E. raffinosus* mega-plasmids

Toxin	Antitoxin	System	Gene symbol	Function
**pCX012922 (*E. raffinosus* CX012922)**				
	MazE antitoxin	Type II TA system	*mazE*	SpoVT / AbrB like domain
	AbiEi_4	Type IV TA system	*abiGI*	Transcriptional regulator, AbiEi antitoxin
AbiEii		Type IV TA system	NA	Nucleotidyl transferase AbiEii toxin
ABC transporter		Type IV TA system	NA	AAA domain, putative AbiEii toxin
ABC transporter		Type IV TA system	*ecsA_2*	AAA domain, putative AbiEii toxin
ABC transporter		Type IV TA system	XK27_07680	AAA domain, putative AbiEii toxin
AAA_33, Zeta_toxin		Type II (epsilon/zeta) TA system	NA	Protein conserved in bacteria
**pF162_2_1 (*E. raffinosus* pF162_2)**				
ABC transporter		Type IV TA system	*ecsA_2*	AAA domain, putative AbiEii toxin
ABC transporter		Type IV TA system	XK27_07680	AAA domain, putative AbiEii toxin
AAA_33, Zeta_toxin		Type II (epsilon/zeta) TA system	NA	Protein conserved in bacteria
	Phd/YeFM antitoxin	Type II (Phd/YeFM) TA system	NA	Antitoxin component of a TA module
ParE toxin		Type II (RelE/ParE) TA system	NA	ParE toxin
		Type IV TA system	NA	AAA domain, putative AbiEii toxin
**pF162_2_2 (*E. raffinosus* pF162_2)**				
	Epsilon antitoxin	Type II (epsilon/zeta) TA system	NA	Bacterial epsilon antitoxin
Zeta toxin		Type II (epsilon/zeta) TA system	NA	Zeta toxin
YoeB toxin		Type II (YefM/YoeB) TA system	*yoeB*	YoeB-like toxin
	Phd/YeFM antitoxin	Type II (Phd/YeFM) TA system	NA	Antitoxin component of a TA module

The TA systems in each mega-plasmid were identified from the eggNOG-mapper annotation results of the complete nucleotide sequence.

Table S7 The predicted plasmid contigs in the genomes of *E. raffinosus* using three methods

Strain	Preditciton method			Plasmid Proportion (%)
	PlasmidFinder/ Plasmid replicon hits	PlasForest/ Predicted plasmid contigs	mlplasmids/ Predicted plasmid contigs	
CX012922	no hit found	pCX012922	pCX012922	25.84
Isolate_3	Rep1, Rep_trans, RepA_N	FKLT01000532.1,FKLT01000530.1,FKLT01000524.1,FKLT01000522.1,FKLT01000521.1, etc. (146)[a]	FKLT01000258.1,FKLT01000268.1,FKLT01000267.1,FKLT01000377.1,FKLT01000039.1, etc. (153)	17.18
ATCC49464	no hit found	ASWF01000009.1	ASWF01000006.1, ASWF01000007.1	10.91
BIOML_A1	no hit found	WXOO01000041.1,WXOO01000042.1, WXOO01000056.1,WXOO01000061.1, WXOO01000065.1, etc. (8)	WXOO01000003.1,WXOO01000004.1, WXOO01000008.1,WXOO01000009.1, WXOO01000011.1, etc. (29)	29.76
cftri2200	Rep_trans	ATKJ02000061.1,ATKJ02000081.1,ATKJ02000091.1,ATKJ02000096.1,ATKJ02000106.1, etc. (22)	KI392041.1,KI392042.1,KI392045.1,ATKJ02000029.1,KI392047.1, etc. (44)	22.54
Colony537	no hit found	no hit found	no hit found	0
DSM5633	Inc18	NZ_JXLA01000101.1,NZ_JXLA01000102.1,NZ_JXLA01000103.1,NZ_JXLA01000104.1,NZ_JXLA01000105.1, etc. (24)	NZ_JXLA01000002.1,NZ_JXLA01000003.1,NZ_JXLA01000006.1,NZ_JXLA01000011.1,NZ_JXLA01000023.1, etc. (32)	25.07
F162_2	Inc18(pF162_2_1)	pF162_2_1, pF162_2_2	pF162_2_1, pF162_2_2	28.76

K36	Rep_trans	JAASIQ010000053.1,JAASIQ010000054.1,JAASIQ010000065.1,JAASIQ010000076.1,JAASIQ010000077.1, etc. (8)	JAASIQ010000006.1,JAASIQ010000012.1,JAASIQ010000015.1,JAASIQ010000017.1,JAASIQ010000019.1, etc. (35)	25.64
L3_072_123	no hit found	JAHABH010000062.1,JAHABH010000037.1,JAHABH010000072.1,JAHABH010000046.1,JAHABH010000051.1, etc. (6)	JAHABH010000002.1,JAHABH010000006.1,JAHABH010000012.1,JAHABH010000015.1,JAHABH010000017.1, etc. (20)	21.36
MGYG_HGUT_01696	Rep_trans	CABLCA010000017.1,CABLCA010000041.1,CABLCA010000054.1,CABLCA010000061.1,CABLCA010000081.1, etc. (25)	CABLCA010000020.1,CABLCA010000029.1,CABLCA010000011.1,CABLCA010000031.1,CABLCA010000043.1, etc. (50)	20.48
N17	no hit found	JAHLOS010000087.1,JAHLOS010000095.1,JAHLOS010000096.1,JAHLOS010000097.1	JAHLOS010000012.1,JAHLOS010000013.1,JAHLOS010000016.1,JAHLOS010000019.1,JAHLOS010000021.1, etc. (20)	14.87
NBRC_100492	Inc18	BCPU01000071.1,BCPU01000074.1,BCPU01000076.1,BCPU01000081.1,BCPU01000088.1, etc. (25)	BCPU01000009.1,BCPU01000014.1,BCPU01000017.1,BCPU01000019.1,BCPU01000025.1, etc. (40)	20.29
HMSC066C04	Inc18, Rep1	KV818692.1,KV818694.1,KV818700.1,KV818703.1,KV818707.1, etc. (33)	KV818769.1,KV818814.1,KV818714.1,KV818761.1,KV818730.1, etc. (38)	32.56
HMSC14A10	Rep_trans	NZ_KV787221.1,NZ_KV787243.1,NZ_KV787245.1,NZ_KV787246.1,NZ_KV787252.1, etc. (24)	NZ_KV787333.1,NZ_KV787255.1,NZ_KV787274.1,NZ_KV787219.1,NZ_KV787345.1, etc. (37)	21.32
HMSC29A04	Rep_trans	KV786285.1,KV786289.1,KV786291.1,KV786297.1,KV786299.1, etc. (21)	KV786307.1,KV786377.1,KV786305.1,KV786391.1,KV786290.1, etc. (34)	25.7

[a] Number in the brackets represents the contig numbers predicted as plasmid in the draft genome sequences.

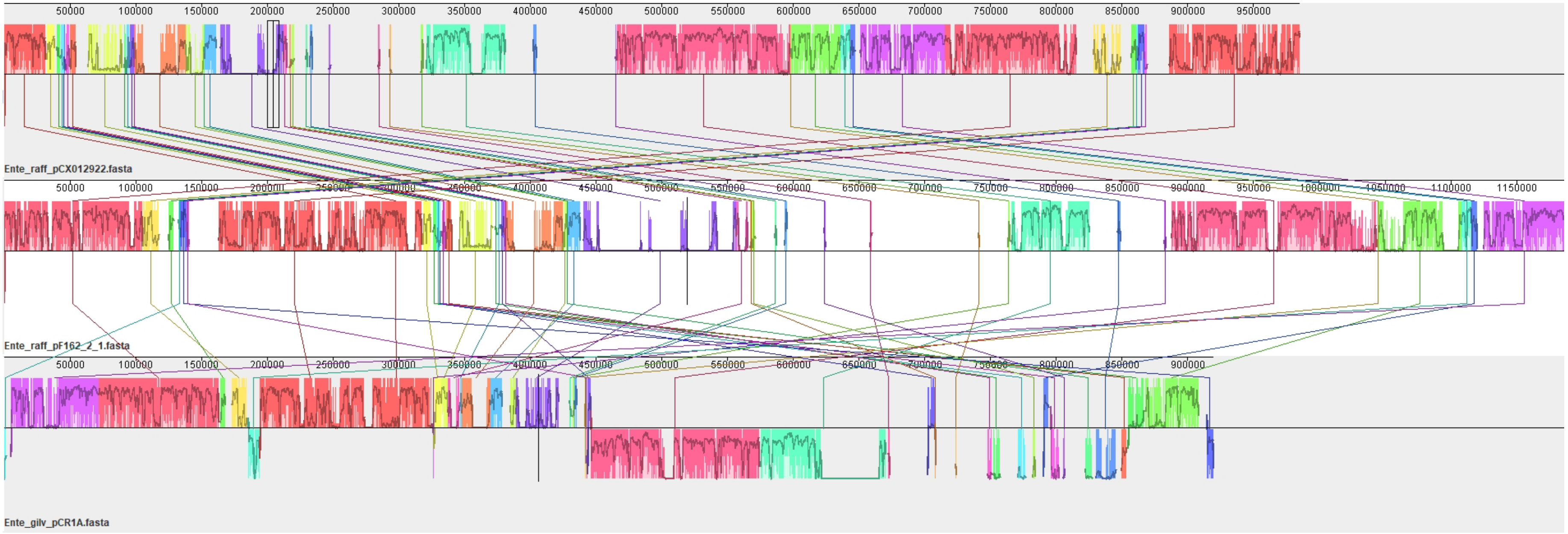

Figure S1. Multiple collinearity between the giant mega-plasmids. Ente_raff_pCX012922.fasta, Ente_raff_pF162_2_1.fasta and Ente_gilv_pCR1A.fasta represent giant mega-plasmids from *E. raffinosus* CX012922, *E. raffinosus* F162_2 and *E. gilvus* CR1 respectively.

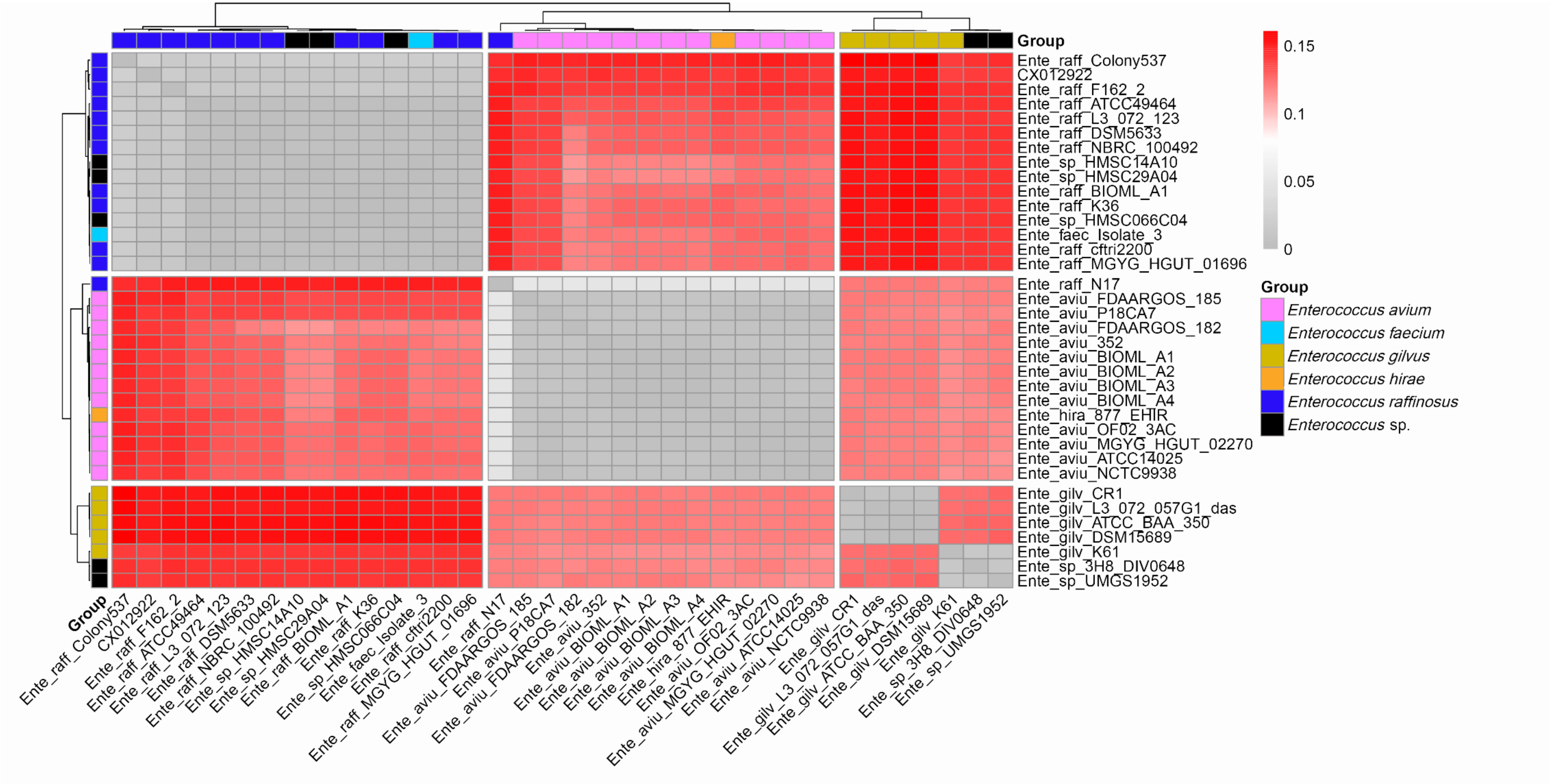

Figure S2. Heatmap displaying the genome distance between the phylogenetically close *Enterococcus* spp. Color bar in the right indicates the genome distance calculated using the "Similar Genome Finder" function in PATRIC.

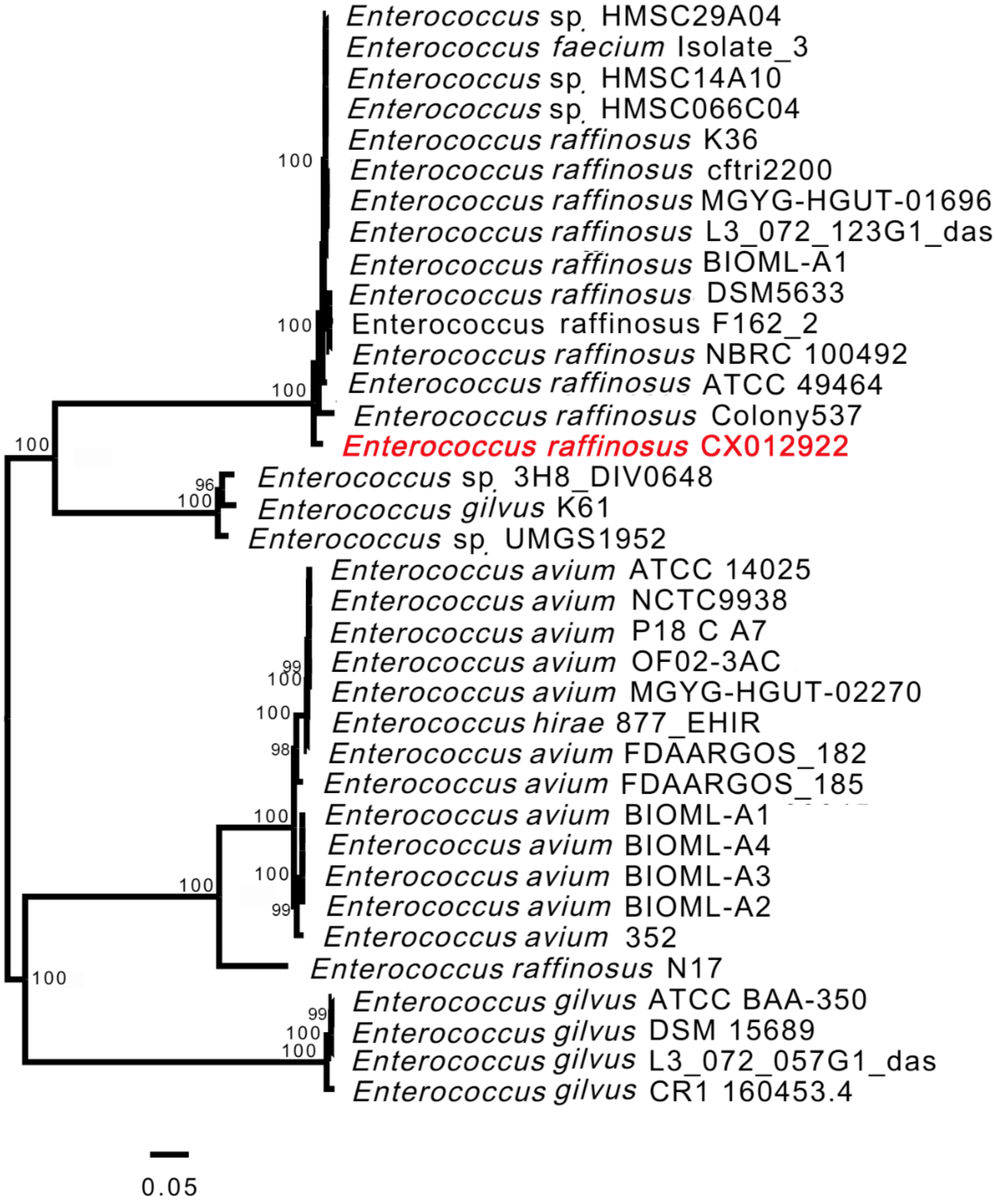

Figure S3. Phylogenomic tree constructed from the whole genome sequences of the strains phylogenetic close to strain CX012922.

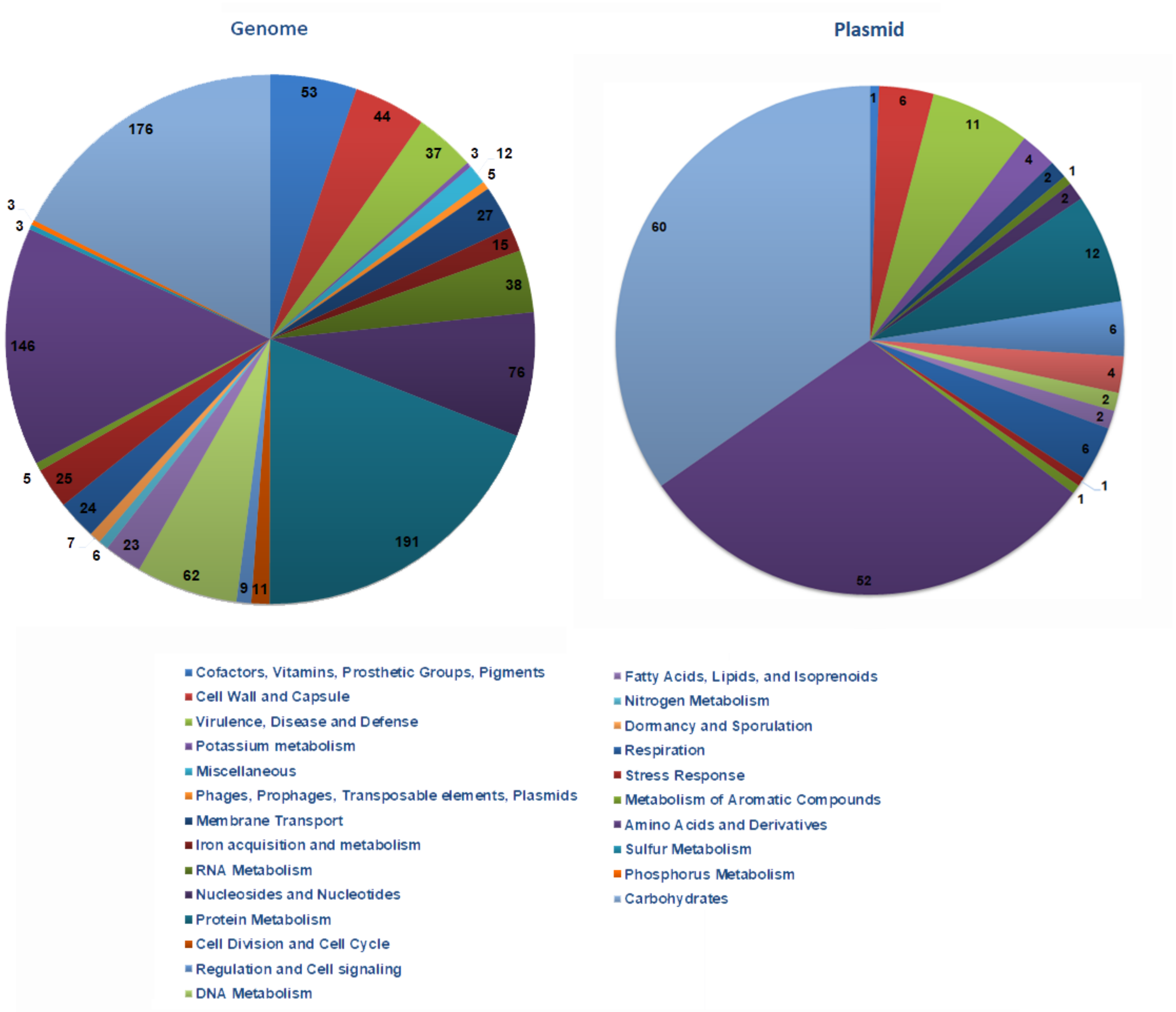

Figure S4. The genome and plasmid genes annotated to SEED subsystem functions.

Reference

[1] Ondov BD, Treangen TJ, Melsted P, Mallonee AB, Bergman NH, Koren S, et al. Mash: fast genome and metagenome distance estimation using MinHash. Genome Biol. 2016;17 (1):1-14.
